# Priorities for efficacy trials of gender-affirming hormone therapy with estrogen: collaborative design and results of a community survey

**DOI:** 10.1007/s42000-024-00532-3

**Published:** 2024-02-05

**Authors:** Shira Grock, Jane Weinreb, Kristen C. Williams, Amy Weimer, Sarah Fadich, Reema Patel, Atara Geft, Stanley Korenman

**Affiliations:** 1 Division of Endocrinology, Diabetes and Metabolism, University of California, Los Angeles, David Geffen School of Medicine, Los Angeles, CA 90095 USA; 2 University of California, Los Angeles, David Geffen School of Medicine, Los Angeles, CA 90095 USA; 3 UCLA Gender Health Program, University of California, Los Angeles, David Geffen School of Medicine, Los Angeles, CA 90095 USA; 4 Division of Endocrinology, Diabetes and Metabolism, VA Greater Los Angeles Healthcare System, Los Angeles, CA 90073 USA; 5 Department of Medicine, University of California, Los Angeles, David Geffen School of Medicine, Los Angeles, CA 90095 USA; 6 Division of General Internal Medicine, VA Greater Los Angeles Healthcare System, Los Angeles, CA 90073 USA

**Keywords:** HRT, GAHT, Estrogen, Transgender, Research design

## Abstract

**Purpose:**

Treatment guidelines for gender-affirming hormone therapy with estrogen (GAHT-E) recommend specific dosing regimens based on limited data. Well-controlled efficacy trials are essential to tailoring treatment to patient goals as the guidelines recommend. The goal of this study was to take a foundational step toward designing community-centered effectiveness trials for gender-diverse individuals seeking GAHT-E.

**Methods:**

Our team developed a cross-sectional survey based on broad clinical experience and consultation with our community advisory board. The survey included 60 items covering demographics, transition history, goals and priorities for treatment, indicators of treatment success, sexual function goals, and future research priorities. The survey was distributed during the summer of 2021, primarily through social networks designed for gender-expansive individuals seeking treatment with estrogen.

**Results:**

A total of 1270 individuals completed the survey. Overall treatment goals most frequently rated “extremely important” or “very important” were the following: (1) improved satisfaction with life (81%), (2) appearing more feminine (80%), (3) appearing less masculine (77%), (4) improved mental health (76%), and (5) being seen as your true gender by others (75%). The three body characteristics most frequently rated “highest priority” or “high priority” among changes were the following: (1) facial hair (85%), (2) breast shape or size (84%), and (3) body shape (80%). The highest-rated research priority was comparing feminization with different routes of estrogen administration.

**Conclusion:**

The goals and experiences of individuals seeking GAHT-E are diverse. Future clinical trials of GAHT-E should be grounded in the needs and priorities of community stakeholders.

## Introduction

Treatment guidelines for gender-affirming hormone therapy (GAHT) recommend dosing regimens with the caveat that treatment should be tailored to patients’ goals; however, there are few data enabling us to ascertain the efficacy of any specific regimen for achievement of patient-centered endpoints [1–3]. Published prospective cohort studies evaluating physical changes achieved via GAHT with estrogen (GAHT-E) offer preliminary evidence of efficacy but are limited by uncontrolled variables [4–7].

Despite the increase in the types of evidence acceptable for regulatory decisions in the USA, the existing evidence remains inadequate for approval of drugs indicated for GAHT. Well-controlled clinical trials are the gold standard for assessing safety, absolute efficacy, and comparative efficacy of treatment but as of March 28th, 2023, a PubMed search revealed only one randomized controlled trial of GAHT [8]. Burinkul and colleagues demonstrated the feasibility of comparative efficacy trials for GAHT but did not measure patient-centered endpoints such as anatomical change or patient-reported outcome measures (PROMs) [8]. This low quality and volume of evidence is widely cited in treatment guidelines [2, 3], systematic reviews [1], and reports from stakeholder engagement events [9].

A foundational problem for building the nascent literature was succinctly summarized in a report from an FDA community listening session as follows: “… participation would be dependent on the goal/endpoint of the [study]. [Participants] noted that there isn’t a common goal shared by people who identify as trans” [10]. Community members are unlikely to participate in research irrelevant to their treatment goals, and trans and gender-expansive communities have widely varying goals. Thus, investigators must navigate the following two essential decisions: (1) defining and describing appropriately homogenous subgroups for assessing physiological changes and (2) defining “efficacy” to reliably and validly represent their treatment goals [11, 12]. This report describes an initial step in answering these questions in collaboration with community stakeholders.

## Methods

Having chosen to focus first on GAHT-E, our research group designed a cross-sectional survey of experiences, expectations, and priorities of individuals seeking GAHT-E in order to prioritize research questions and treatment outcomes. Because few data and no validated survey measures exist on this topic, our research group developed a novel questionnaire based on clinical experience and in consultation with our institution’s community advisory board (CAB). A non-provider team member attended regularly scheduled CAB meetings for advice and feedback. The team subsequently met with two CAB members possessing personal GAHT-E experience to collaboratively review and edit the survey draft, focusing on refining language and broadening the experiences represented; CAB members were paid an honorarium for their time and expertise.

### Population definition

Our research group sought to prioritize affirmation, concision, clarity, and precision in defining the target population. Our goal was to specify a group likely to have relatively similar endogenous hormone environments and treatment responses while affirmatively including individuals of varied identities and experiences. Our study thus focused on the chosen treatment modality over the inherent characteristics of participants by defining the primary inclusion criterion as “seeking GAHT-E.” Gender identity and GAHT-E were not referred to as “feminizing” to avoid assumptions or implications of binary gender. Instead, GAHT-E was defined as “medications taken for the purpose of altering your body toward body characteristics such as fat distribution across the body, breast growth, and finer body hair. This can include medications like estrogen, spironolactone, or others.”

CAB input and clinical experience indicated that defining this population by natal sex inherently invalidated their lived identities; thus, our research group sought to develop a population definition without reference to natal sex. Our team was unable to develop another approximation of endogenous hormone environment that was as clear and concise—though lacking precision—as the widely used “sex assigned at birth.” CAB members suggested “sex listed on your original birth certificate,” which was abbreviated to “listed ‘male’ at birth” or “LMAB,” hoping its similarity to the established term “assigned male at birth” or “AMAB” would enhance clarity while reducing invalidating and oppressive connotations.

Our final population definition included individuals listed as “male” on their original birth certificates who were 18 years of age or older and seeking GAHT-E. For initial communication (e.g., flyers), the target population was described as “treatment-seeking trans and gender-expansive people listed as ‘male’ at birth.” GAHT-E was not specified for the sake of concision, reasoning that GAHT-E experience or interest would, in any case, be common among respondents. Additionally, the research group did not want to exclude individuals interested only in other treatments, including androgen blockade monotherapy or surgical therapies.

### Survey design

This study was reviewed and approved by the UCLA South General Institutional Review Board. Our survey of 60 items covering demographics, transition history, treatment goals and experiences, and research priorities was distributed using Qualtrics, an online survey tool. As an incentive and token of gratitude, individuals could enter a raffle for one of eight gift cards regardless of participation. To prevent linking survey responses to identifiable raffle entries, a separate entry form accessible only through a restricted link provided upon completion or declination of the survey was created.

Prior to any questions, the electronic survey presented study information, including a statement that participation was voluntary and an item requesting consent to continue. To ensure respondents met inclusion criteria, three initial screening items excluded respondents who (1) did not consent, (2) were younger than 18 years of age, or (3) were listed as “female” or “intersex” on their original birth certificates. Built-in tools of the Qualtrics platform were utilized to discourage multiple participation and bot responses as well as to remove identifiers, such as IP address, from the final dataset.

Item text and response options were based on personal experiences, patient reports, and CAB consultation. Given the paucity of existing data to guide scale development, face-valid single items were created to be analyzed individually. Several items included an additional free-text option to solicit additional information where available response items were insufficient. For affirmation and brevity, logic structures were used to avoid displaying questions not applicable to the respondent. For example, participants were asked if they had a penis prior to questions about erectile function, which were then displayed only following an affirmative response. Broadly inclusive definitions of terms were utilized (e.g., defining “sex” as “any activity you use for erotic stimulation whether you are alone or with another person. This includes masturbation, oral sex with a partner, penetrative sex, and a wide range of other activities”).

In contrast, some items were designed restrictively to simplify future analyses. For instance, the complex reality of gender identity was balanced with the statistical simplicity of finite, mutually exclusive choices with the item “With which of these gender identities do you *most* identify?” Insight on item acceptability and utility was sought through the “a gender not listed here” option, which included a free-text field.

### Recruitment

The survey was distributed as follows: by (1) emailing patients who previously agreed to receive research opportunities, (2) sharing survey flyers with community allies, and (3) social media and community forum posts. By utilizing our community connections and prioritizing online outreach, the study team hoped to reach a more diverse convenience sample than our institution’s primarily white, non-Hispanic, and privately insured population [13]. A team member engaged with relevant online communities led an outreach effort focused on groups created by and for treatment-seeking individuals using relevant terms (e.g., “MtF,” “transfeminine,” and “GAHT”).

### Analysis

Using SAS version 9.4 (SAS Institute, Cary, NC, USA), data was examined for anomalous response patterns such as duplicate responses and outliers. Once data was determined to be free of such artifacts, built-in analysis and reporting tools on the Qualtrics platform were used to produce descriptive statistics. Given our descriptive goal, the study did not include *a priori* hypotheses or conduct significance tests. Instead, we sought to describe response variance and free-text additions. Categorical responses are reported as proportions of responders to respective individual items. For items with many free-text responses, answers were categorized by simple topic (e.g., body part described) and reported by number of respondents addressing each topic. The recommendations in the Checklist for Reporting of Survey Studies (CROSS) were followed in developing this report [14].

## Results

**Fig. 1:**
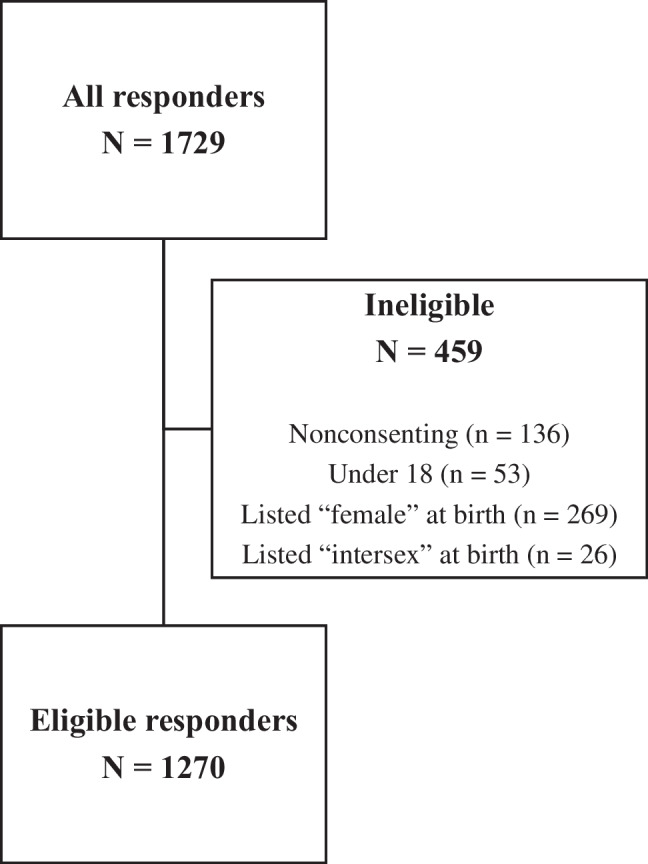
Flow diagram for inclusion and exclusion of participants. Note that exclusion criteria were nonexclusive

Of 1729 individuals who accessed and responded to at least one screening or consent item, 1270 eligible individuals (73%) completed the survey between June 8 and July 31, 2021. Details for participant eligibility are displayed in Fig. 1.

### Demographics

Respondent demographics are presented in Table 1. Though 90% of respondents reached the survey via social media, our recruitment strategy generally did not meet our goal of a sample more diverse than that of our local program. Predominantly, respondents were white (77%), under age 35 (84%), and residing in the USA (81%).

**Table 1:** Demographic characteristics of the study sample (*N* = 1270)

	%	*n*
Age		
18–25	41.6	528
26–35	42.4	539
36–45	9.5	121
46–55	3.2	40
56–65	2.2	28
Most congruent gender identity		
Female	24.2	307
Transgender female	45.0	572
Male	19.6	249
Transgender male	4.3	55
Genderqueer	0.8	10
Genderfluid	1.2	15
Non-binary	3.5	45
A gender not listed here (please specify)	1.1	14
Agender	0.2	3
Location		
United States-West	32.7	410
United States-South	20.3	255
United States-Midwest	15.0	188
United States-Northeast	13.0	164
North America-Canada	4.7	60
North America-Mexico	0.5	6
Europe	8.8	111
Africa	0.3	4
Asia	0.7	9
South America	0.4	5
Australia	3.3	41
Education		
Less than high school diploma	2.5	32
High school graduate (high school diploma or equivalent including GED)	16.7	209
Some college but no degree	26.7	334
Associate degree in college (2-year)	15.5	193
Bachelor’s degree in college (4-year)	27.4	344
Master’s degree	7.0	87
Doctoral degree	2.4	30
Professional degree (JD, MD)	1.6	20
Racial identity		
Asian	3.9	51
Native Hawaiian or Pacific Islander	2.6	34
Other	2.0	26
Black or African American	6.4	84
White	77.3	1019
Native American or Indigenous	7.9	104
Spanish, Hispanic, or Latinx		
Yes	21.0	257
No	79.0	966
Insurance coverage		
Yes, I have insurance through my employer	27.5	344
Yes, I have insurance that I pay for myself	28.2	353
Yes, I have state or nationally sponsored coverage such as Medicare or Medicaid	24.6	308
No, I do not have medical coverage	5.5	69
I’m not sure if I have medical coverage	2.1	26
Yes, I have coverage through my parents	12.2	153
Exposure to hormone therapy		
Yes	81.2	1017
No	18.4	229
More than a year	54.5	678
Less than a year	45.5	566

Regarding gender identity, one respondent commented that “transgender female” did not make sense because “female” is a sex category, while “woman” is a gender category. Another respondent also added “transgender woman” as free text. Overall, 14 individuals (1%) selected “a gender not listed here.” Of associated free-text responses, only “demigirl,” listed twice, was listed more than once.

### Treatment goals

**Fig. 2:**
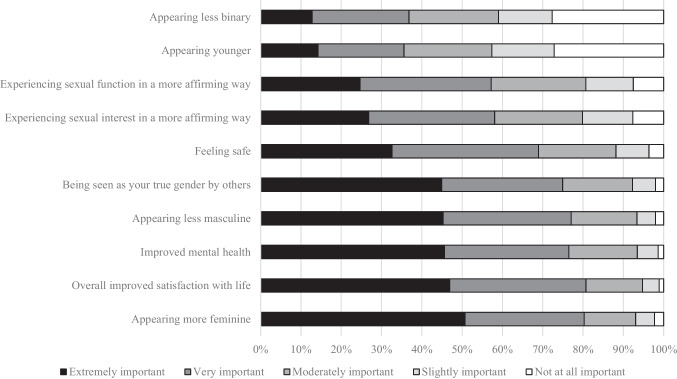
Goals when starting gender-affirming treatment

**Fig. 3:**
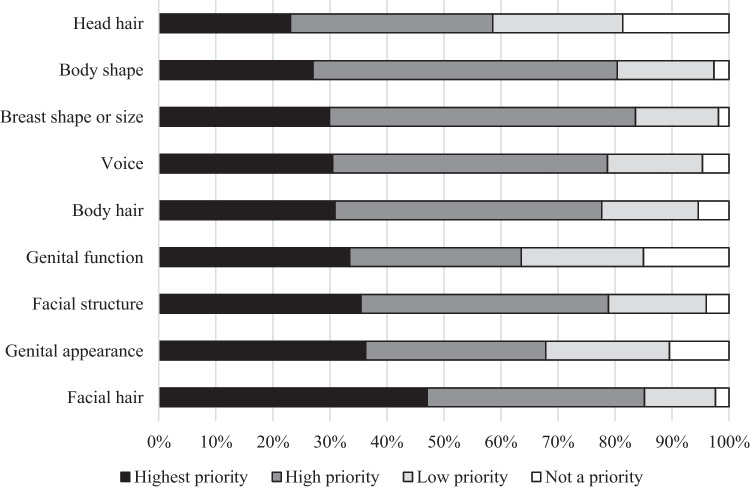
Priorities for physical change

Figure 2 displays the overall goals of gender-affirming treatment. Treatments are listed in order of the frequency with which they were rated “extremely important.” Similarly, physical features prioritized for change are listed in Fig. 3. Free-text responses are described in Table 2.

**Table 2:** Additional features noted as priorities for change in free-text responses

Feature	Number of respondents
Acne or other skin concerns	28
Hands, feet, or both	21
Adam’s apple or trachea	16
Shoulder, chest, or ribcage shape	15
Overall body shape	11
Muscle mass	11
Height	11
Legs or thighs	10
Emotional, psychological, or behavioral changes	10
Hips or buttocks	9
Facial features	8
Weight	6
Odor	5

### Subjective efficacy indicators

Table 3 lists potential experiences during GAHT-E in the order of frequency endorsed as indicators as to whether GAHT-E was working or not. Free-text positive indicators added by multiple respondents included qualitative changes to sexuality, changes in body odor, changes to face shape, and reduced head hair loss. Additions to negative indicators were similar to (e.g., changes in body odor) or opposite of (e.g., head hair loss) positive indicators.

**Table 3:** Subjective indicators of efficacy

	%	Count
If your treatment WAS WORKING the way you want, which of these would be important indicators of this? (multiple responses allowed)		
Breast growth	76.27	871
Body shape changes	71.89	821
Decreased body/facial hair	68.39	781
Skin softening	63.49	725
Emotional state	56.48	645
Decreased erectile function	28.46	325
Increased sex drive	16.46	188
Decreased sex drive	14.10	161
Increased erectile function	8.23	94
Other	2.36	27
Total (*n*)		1142
If your treatment was NOT WORKING the way you want, which of these would be important indicators of this? (multiple responses allowed)		
Emotional state	47.20	522
Skin changes	46.56	515
Hair thickening	42.86	474
Increased sweating	40.14	444
Increased erectile function	36.53	404
Increased sex drive	29.75	329
Decreased erectile function	14.74	163
Decreased sex drive	11.75	130
Other	3.80	42
Total (*n*)		1106

### Research priorities

From a list of potential research questions, respondents selected up to three as the most important. These are listed in Table 4 by proportion of respondents endorsing them. Research questions added in free-text responses were similar in their focus on assessing efficacy and safety, additionally suggesting comparing different types of estrogen, testing estrogen monotherapy, and measuring head hair loss as an outcome.

**Table 4:** Research question priorities (respondents could select up to three priorities)

	%
Which administration route (patch, injection, pills) of hormone therapy is most effective at feminizing the body?	54.32%
Which administration route (patch, injection, pills) of hormone therapy has the fewest side effects and safety concerns?	43.01%
Does progesterone help with feminizing the body?	36.87%
Do higher levels of estrogen in the blood lead to more feminization or faster feminization?	36.70%
What is the most effective way to block testosterone production or action?	30.74%
Which hormone treatment regimen maintains sexual function most effectively?	18.31%
What is the best way to treat hormone therapy-related painful erections and penile atrophy?	15.63%
How does diet interact with hormone therapy?	13.73%
How does hormone therapy interact with other medications?	13.39%

## Discussion

This study emphasizes the immense variance in identities, experiences, and treatment goals among individuals seeking GAHT-E. For example, although respondents rated head hair as a “low priority” or “not a priority” more frequently than any other feature, nearly 60% still rated it as “highest priority” or “high priority.” Furthermore, reducing head hair loss was the only specific endpoint listed as a research priority in free-text responses. Similarly, while 16% of individuals felt that *increased* sex drive was a sign of treatment efficacy, 14% reported that *decreased* sex drive was a sign of efficacy. Speaking to the complexity of gender identity, 24% of our respondents identified as male or transgender male, and 87% of that group reported current or former use of GAHT-E compared to 82% of the entire sample. Their GAHT-E use indicates that this group was appropriately included and that centering inclusion criteria on treatment choice allowed us to recruit a group who may not have responded to identity-based recruitment or may have been excluded by identity-based screening items.

Just as informative are the commonalities in our data. Current guidelines for GAHT-E recommend matching treatment regimens with individualized goals, but research evaluating *how* to personalize treatment is lacking [2, 3]. Respondents’ highest-rated research priority was comparing routes of estrogen administration in facilitating feminization. Among known outcomes of GAHT-E, respondents most prioritized breast growth, facial and body hair reduction, and body shape changes. The highest prioritized overall treatment goal was “satisfaction with life.” These data provide a foundational step toward incorporating the needs of the community into future research and patient care by roughly outlining which treatment goals should be prioritized when designing efficacy studies. While a few prospective cohort studies have attempted to assess some of these differences in physical changes with varying levels or routes of estrogen [4–6], the results have been limited by uncontrolled variables. Active-control trials can provide vital information by varying a single aspect of treatment (e.g., route of administration).

An expansive review of methods to quantitatively assess prioritized outcomes of GAHT-E is outside the scope of this article; however, it should be noted that one substantial barrier to efficacy trials is that most available measures that were developed with cisgender samples have not been validated in gender-diverse populations, and when used have often been found to be of mixed utility in assessing GAHT-E effects [15–20]. To cite an example, most methods that evaluate breast growth assess volume, which translates easily as a continuous variable for analysis but may not represent patient preferences for overall breast shape [21]. Measurement tools should be grounded in the needs of the relevant population and thoroughly capture the patient experience [22]. The GENDER-Q, for instance, is a modular set of PROMs currently in field testing after development in collaboration with gender-diverse stakeholders which promises to be ideal for GAHT trials [23]. The GENDER-Q may be an ideal model to help update existing anatomical measurement tools to become more patient-centric.

Acknowledging diverse treatment goals is also a requisite for providing an informed, individualized approach to patient care. To illustrate, individuals who view increased libido as a sign of treatment failure may benefit from aggressive testosterone suppression. Conversely, individuals who want to maintain erectile function and libido may benefit from a liberal testosterone goal and early conversations about phosphodiesterase inhibitors. While it is not clear that adjusting testosterone goals directly affects libido, approaching treatment with an open mind, querying specific goals, and allowing patients room to explore such possibilities may improve the doctor-patient relationship, empower patients to be active participants in their care, and ultimately help providers gain understanding of ways to personalize treatment.

Eliciting patient goals at an initial intake visit can also elucidate which adjunctive therapies to utilize. For example, those who rate body hair reduction as a top priority should be referred for laser or electrolysis early in transition, while those who prioritize voice change warrant an early referral for voice therapy. Similarly, if androgenic alopecia is a large source of dysphoria, it may be worthwhile discussing the addition of minoxidil to initial hormone therapy. It is also important for providers to be mindful that not all individuals with gender incongruence desire hormone therapy. Discussing specific treatment goals may reveal that utilizing exclusively non-hormonal treatments such as scalp hair restoration surgery, laser hair removal, and voice therapy is more suitable for some.

## Limitations

This report describes methods and findings of initial steps toward designing efficacy trials for GAHT and as such is limited in its generalizability. Because the survey was developed based on patient-reported experiences, personal experiences, and the advice of our CAB, our decisions were substantially shaped by our locality and the patients seen at our center. The study team had hoped to reach a broader population via global recruitment within online communities to assess broader validity, but ultimately our sample was substantially similar to those individuals seen in our clinics.

Although it speaks to the efficacy of our recruitment efforts, having 90% of participants recruited from social media may bias our results. Social media offer important spaces for building transgender, nonbinary, and other gender-expansive communities [24–27], but as with physical spaces, access is not always equitable [28, 29]. Further, the online groups from which survey respondents were recruited often focused on *starting* gender-affirming treatments, likely contributing to our sample’s relatively short duration of hormone therapy. The groups frequently included such terms as “MtF” or “transfeminine” in titles and descriptions and may have appealed less to nonbinary users; only 3.5% of our respondents reported identifying most with a nonbinary identity, while the Williams Institute estimated that 32.1% of transgender adults in the USA identify as non-binary [30].

Future studies should more directly target communities that our study left out, preferably via qualitative methods that allow participants greater freedom to express experiences different from those encoded in a structured questionnaire. Additionally, the majority of our study participants were aged 18–35. Future studies should target more specific age ranges to assess whether age affects goals and expectations. Our findings represent only a starting point for characterizing community research priorities; different methods are essential to broadening and deepening our understanding.

## Conclusions

Well-controlled efficacy trials will be essential to gaining regulatory approval for the specific use of GAHT medications, refining clinical care guidelines, tailoring individual treatment plans, and ultimately understanding how to safely and effectively support gender-diverse patients. To best do so, trial designs must be grounded in the needs, priorities, and experiences of actual GAHT users. Based on our collaboratively developed community survey, the medical community should prioritize trials comparing estrogen administration routes in their effects on breast growth, body hair, body shape, and overall well-being. Our findings can serve as a starting point for GAHT-E trials and our methods as one approach to meeting the challenge of their design.

## Data Availability

Data supporting the findings in this report are fully anonymous and available upon reasonable request to the corresponding author.
